# The Earth Microbiome project: successes and aspirations

**DOI:** 10.1186/s12915-014-0069-1

**Published:** 2014-08-22

**Authors:** Jack A Gilbert, Janet K Jansson, Rob Knight

**Affiliations:** Institute for Genomics and Systems Biology, Argonne National Laboratory, Lemont, IL 60439 USA; Department of Ecology and Evolution, University of Chicago, 5640 South Ellis Avenue, Chicago, IL 60637 USA; College of Environmental and Resource Sciences, Zhejiang University, Hangzhou, 310058 China; Pacific Northwest National Laboratory, PO Box 999, MSIN: J4-18, Richland, WA 99352 USA; Department of Chemistry and Biochemistry and BioFrontiers Institute, University of Colorado, Boulder, CO 80309 USA; Howard Hughes Medical Institute, Boulder, CO 80309 USA

The Earth Microbiome Project (EMP) was launched in August 2010, with the ambitious aim of constructing a global catalogue of the uncultured microbial diversity of this planet. The primary vision of the Earth Microbiome Project, to process the microbial diversity and functional potential from approximately 200,000 environmental samples, marks it as an undertaking so massive that it was at first considered to be pure folly (as late as 2012, Jonathan Eisen was quoted in *Nature* as saying ‘Knight and Gilbert literally talk about sampling the entire planet. It is ludicrous and not feasible - yet they are doing it’ [1]).

The initial concept arose out of a Department of the Environment (DOE) sponsored workshop on the promise of terabase-scale sequencing in Snowbird, Utah, designed to inspire research ideas using new technology to revolutionize microbial ecology and our understanding of the microbial world [2]. Many other exciting projects also evolved from that meeting, including efforts to extend the sequencing of type strains of cultured bacterial taxa, which in itself has become the Microbial Earth Project [3]. In October 2010, EMP pioneers held a small workshop at Argonne National Laboratories to determine the most effective way to jumpstart such an initiative. At this meeting, we agreed that the only feasible route to acquire and process 200,000 samples was through crowdsourcing, soliciting donations of samples from researchers around the world. This was identified as a key flaw in the design, on the grounds that it would not be possible to convince researchers to part with samples that had been painstakingly collected for inclusion in a single effort [4]. Fortunately, the participants’ generosity has greatly exceeded what we could have hoped for, and the crowdsourcing approach has been a success.

We floated this strategy initially as a potentially viable approach based on the precedent of existing programs that followed broadly similar designs, especially the International Census of Marine Microbes [5] and the Human Microbiome Project [6]. The basic design was founded on the principle of coordinated sample collection, and standardization of contextual metadata acquisition, DNA extraction, PCR and amplicon and shotgun sequencing approaches, and an open-source analytical platform with free, unrestricted access to both the amplicon and metadata immediately following completion of the analysis. Initially the effort was funded primarily by unrestricted funds available to the principle investigators through Argonne National Laboratory, Lawrence Berkeley National Laboratory, the Howard Hughes Medical Institute, and donations from corporate sponsors. Under this effort, the Earth Microbiome Project committee developed the standard protocols [7], contacted and collaborated with researchers from numerous different microbial ecology disciplines, from human, animal, plant, terrestrial, marine, freshwater, sediment, air, built environment and every intersection of these ecosystems. By August 2012, less than 2 years since its initiation, the Earth Microbiome Project had processed approximately 7,000 environmental samples, generating 16S rRNA amplicon data and releasing these data using an open portal through the Quantitative Insights into Microbial Ecology (QIIME) database. In June 2013, the EMP received awards from the WM Keck Foundation and the John Templeton Foundation to support activities to bring the catalogue up to 50,000 samples processed, and as of July 2014 we have reached over 30,000 (compared with the phase 1 Human Microbiome Project amplicon analysis of 5,771 samples [8]). In its planning phase, the EMP proposed the co-analysis of samples using metagenomics and metabolic modeling of ecosystems, and these aims are still viable, but such efforts have to date been more targeted to specific environments and studies. As it stands, the EMP represents the largest effort to characterize the diversity, distribution, and structure of microbial ecosystems across the earth, achievable only through coordinated collaboration of all of the independent research projects (166) that comprise the EMP. Although each hypothesis-driven study provided by our collaborators can tell its own story, the real power of the EMP is through meta-analysis of these data, empowering researchers to develop and use samples acquired from myriad ecosystems to test hypotheses in microbial ecology. Importantly, this pooled data resource also provides an unparalleled opportunity to contextualize individual studies by defining the patterns they see in a global context. These large-scale meta-analyses can enable researchers to ask unique questions regarding the biogeography, dynamic dispersal, and ecology of the microbial planet.

## Current studies, ecosystem coverage, and immediate observations

In the currently available EMP database (as of July 2014) [9] there are samples acquired from >200 collaborators, comprising more than 40 different biomes, defined for broad categories including marine pelagic water, freshwater lake sediment, human-associated, and so on. At a ‘30,000 feet’ perspective the EMP is identifying the environmental characteristics that correlate with microbial community structure within and between these different biomes. However, as the EMP is a collection of individual projects, each with a core hypothesis, it is also possible to discuss the immediate observations associated with individual studies. For example, exploration of human saliva from obese versus normal-weight individuals showed that while saliva was able to alter the aromatic properties of wine, only a few microbial taxa were likely to be responsible for this [10]. This preliminary study shows that oral microbes may influence the aromatic properties of food and drink, altering our satiation response. In soil systems, microbial communities from prairie soils across the Midwest of the United States of America were sequenced by the EMP. This ecosystem has been mostly replaced through agricultural land-use, and this study showed that the major shifts in their composition are driven almost exclusively by the changing relative abundance of Verrucomicrobia and its influence on carbon dynamics [11]. These analyses could be useful in helping improve prairie restoration efforts. In deep soil samples from the Russian permafrost, the EMP characterized microbial communities associated with buried organic matter, helping to identify the bacteria that were degrading the soil organic matter in these systems [12]. In deep-sea sediments from the Gulf of Mexico, the EMP data have provided understanding of how the microbial communities responded to the oil pollution from the Deepwater Horizon Oil Spill [13,14]. Another example of investigating human impact is the analysis of freshwater river sediments along a gradient of human influence, whereby the EMP data on the microbial communities demonstrate impact-specific signals [15]. The diversity of study sites and research questions embedded in these first 30,000 samples is extraordinary, yet this is just the tip of the iceberg. Initial analysis of 10,000 of the samples identified approximately 6 million bacterial taxonomic units (genus or species level taxa), only a small fraction of which could be mapped to known phylogenies using 16S rRNA databases such as GreenGenes [16]. The frequency and distribution of these species can enable us to address interesting questions, for example, regarding the distribution of taxa across different soil ecosystems; the EMP datasets suggest that there is considerable overlap in taxa between sites, with organisms that are abundant at one location being extremely rare in another location, as previously demonstrated from marine sites [17].

A small number of concerns regarding the existing data have been raised by communities focusing on specific systems or taxa. For example, as with all studies using PCR, there are biases associated with the EMP PCR primers: they are not efficient at amplifying marine *Pelagibacter ubique* targets. As a result, new primers have been designed that should be more efficient in amplifying *Pelagibacter*, an important taxon in marine systems; however, we need to determine how efficient these new primers will be at amplifying all the other bacteria from other environments. As such, a study is underway to investigate whether rescuing *Pelagibacter* has deleterious consequences for other taxa or systems. However, because DNA extraction protocols themselves can have different biases depending on the environmental matrix from which the DNA is extracted [18], and PCR reagents can have contaminants that may influence amplification [19], the number of potential biases that could influence analysis is large and the key for cross-system analyses is consistent protocols. We are taking all sensible precautions to catalogue and determine potential biases: by recording all procedural and analytical variables it will be possible to determine which specific protocol elements may influence interpretation and whether the effects of these technical sources of variation limit our ability to identify important factors structuring microbial diversity.

## Creating an EMP operation taxonomic unit table

One major challenge has been creating a master table delineating the abundance of each type of organism in each environment. With 7,000 samples for the Shenzhen meeting in 2011 [20], existing tools could barely handle the data load. In particular, the operation taxonomic unit (OTU) table, which converts the raw sequence data into a sample-by-OTU table giving the taxon abundances, strained the limits of what could be done in the traditional ‘dense’ format in which there is a slot for the abundance of each possible taxon in each environment, even if that slot has a zero count. Simply loading the table into memory and accessing specific taxa or samples became impossible as the dataset grew. Accordingly, we developed the Biological Observation Matrix (BIOM) file format [21], which reduced an early version of the EMP OTU table (6,164 samples by 7,082 OTUs) from 175 MB to 12 MB. Further improvement has been achieved by the recent move in BIOM 2.1 to HDF5, a file format used widely by physicists, climate scientists, and others needing random access to subsets of vast files. With these improvements, which are being developed fully open-source on the github repository [22], we expect that interested parties will be able to manipulate the full EMP OTU table on their laptops rather than requiring large-scale compute resources.

There are many different methods for analyzing the sequence data to obtain clusters of related sequences, each with advantages and drawbacks. For example, clustering sequences *de novo* produces a gold standard sequence cluster (a robust classification of a taxonomically similar group of sequences), but is very slow, while a reference-based protocol, where sequences are matched in a phylogenetic tree, is very fast but throws out sequences that fail to hit a reference. Another important challenge is visualization. QIIME [23] is the analysis architecture primarily used by the EMP, and it has long relied on KiNG [24], a molecular graphics package, for producing three-dimensional principal coordinates plots, essentially treating the community locations as atoms in a very curious molecule. However, as the size of the EMP dataset continued to grow, and the environmental contextual data became richer, the strategy of creating different views of the dataset colored by each field of contextual data (for example pH, dissolved organic carbon, and each of the hundreds of other variables captured by samples in the EMP) became unwieldy. To overcome these challenges, and to provide a three-dimensional graphics component that is directly embeddable in current web technologies, we developed EMPeror [25], software that uses current web standards such as HTML5 and OpenGL, to display even vast datasets and to explore and to recolor them dynamically.

## The future

The EMP will continue to grow and adapt as new collaborators and new technologies are added. Generating the taxon matrix in BIOM format for the existing 30,000 samples will help us to provide advice on the biomes and questions that should be targeted for the next 20,000 samples. We are also exploring metagenomic analyses for studies where the data can be used to test hypotheses regarding the ecology of microbial metabolic function (for example, [11,13,15]). At present, metagenomic data associated with individual studies have been made available through traditional routes (EBI, NCBI submissions), but we are working towards explicit submission and analysis pipelines for these data, including downstream analyses such as genome assemblies and metabolic pathway reconstruction. The success of the EMP has been in generating a coordinated exploration of the microbial world, and in providing the facility for data generation to collaborators who previously did not have such capacity. Primarily this has been achieved through the generation of open access data and analysis platforms that facilitate interpretation. As we move forward, we will continue to explore new avenues for collaboration, including potentially going beyond the Earth to explore extra-terrestrial locations.
